# Supplementary material for: The impact of the route of administration on the efficacy and safety of the drug therapy for patent ductus arteriosus in premature infants: a systematic review and meta-analysis
Source: PeerJ. 2024 Jan 29;12:e16591. doi: 10.7717/peerj.16591 (PMC10832619; doi:10.7717/peerj.16591)
Supplement: Supplemental Information 1 [file peerj-12-16591-s001.pdf]

Supplementary Material

Appendix 1 Search strategy

("Premature Birth"[MeSH Terms] OR ("Premature Birth"[MeSH Terms] OR ("premature"[All Fields] AND "birth"[All Fields]) OR "Premature Birth"[All Fields] OR ("birth"[All Fields] AND "premature"[All Fields]) OR "birth premature"[All Fields] OR ("Premature Birth"[MeSH Terms] OR ("premature"[All Fields] AND "birth"[All Fields]) OR "Premature Birth"[All Fields] OR ("premature"[All Fields] AND "births"[All Fields]) OR "premature births"[All Fields]) OR ("Premature Birth"[MeSH Terms] OR ("premature"[All Fields] AND "birth"[All Fields]) OR "Premature Birth"[All Fields] OR ("preterm"[All Fields] AND "birth"[All Fields]) OR "preterm birth"[All Fields]) OR ("Premature Birth"[MeSH Terms] OR ("premature"[All Fields] AND "birth"[All Fields]) OR "Premature Birth"[All Fields] OR ("birth"[All Fields] AND "preterm"[All Fields]) OR "birth preterm"[All Fields]))) AND ("ductus arteriosus, patent"[MeSH Terms] OR ("ductus"[All Fields] AND "arteriosus"[All Fields] AND "patent"[All Fields]) OR "patent ductus arteriosus"[All Fields] OR ("patent"[All Fields] AND "ductus"[All Fields] AND "arteriosus"[All Fields]) OR ("ductus arteriosus, patent"[MeSH Terms] OR ("ductus"[All Fields] AND "arteriosus"[All Fields] AND "patent"[All Fields]) OR "patent ductus arteriosus"[All Fields] OR ("patency"[All Fields] AND "ductus"[All Fields] AND "arteriosus"[All Fields]) OR "patency of the ductus arteriosus"[All Fields]) OR ("ductus arteriosus, patent"[MeSH Terms] OR ("ductus"[All Fields] AND "arteriosus"[All Fields] AND "patent"[All Fields]) OR "patent ductus arteriosus"[All Fields] OR ("patent"[All Fields] AND "ductus"[All Fields] AND "arteriosus"[All Fields] AND "familial"[All Fields])) OR "ductus arteriosus, patent"[MeSH Terms]) AND ("Drug Therapy"[MeSH Terms] OR ("Drug Therapy"[MeSH Terms] OR ("drug"[All Fields] AND "therapy"[All Fields]) OR "Drug Therapy"[All Fields] OR ("therapy"[All Fields] AND "drug"[All Fields]) OR "therapy drug"[All Fields] OR ("Drug Therapy"[MeSH Terms] OR ("drug"[All Fields] AND "therapy"[All Fields]) OR "Drug Therapy"[All Fields] OR ("drug"[All Fields] AND "therapies"[All Fields]) OR "drug therapies"[All Fields]) OR ("Drug Therapy"[MeSH Terms] OR ("drug"[All Fields] AND "therapy"[All Fields]) OR "Drug Therapy"[All Fields] OR ("therapies"[All Fields] AND "drug"[All Fields]) OR "therapies drug"[All Fields]) OR ("chemotherapy s"[All Fields] OR "Drug Therapy"[MeSH Terms] OR ("drug"[All Fields] AND "therapy"[All Fields]) OR "Drug Therapy"[All Fields] OR "chemotherapies"[All Fields] OR "Drug Therapy"[MeSH Subheading] OR "chemotherapy"[All Fields]) OR ("chemotherapy s"[All Fields] OR "Drug Therapy"[MeSH Terms] OR ("drug"[All Fields] AND "therapy"[All Fields]) OR "Drug Therapy"[All Fields] OR "chemotherapies"[All Fields] OR "Drug Therapy"[MeSH

Subheading] OR "chemotherapy"[All Fields]) OR ("Drug Therapy"[MeSH Terms] OR ("drug"[All Fields] AND "therapy"[All Fields]) OR "Drug Therapy"[All Fields] OR "pharmacotherapies"[All Fields] OR "Drug Therapy"[MeSH Subheading] OR "pharmacotherapy"[All Fields]) OR ("Drug Therapy"[MeSH Terms] OR ("drug"[All Fields] AND "therapy"[All Fields]) OR "Drug Therapy"[All Fields] OR "pharmacotherapies"[All Fields] OR "Drug Therapy"[MeSH Subheading] OR "pharmacotherapy"[All Fields])) OR ("Ibuprofen"[MeSH Terms] OR ((("alpha methyl 4"[All Fields] AND "2-methylpropyl"[All Fields]) AND ("benzeneacetic"[All Fields] AND ("acids"[MeSH Terms] OR "acids"[All Fields] OR "acid"[All Fields]))) OR ("Ibuprofen"[MeSH Terms] OR "Ibuprofen"[All Fields] OR "ibumetin"[All Fields]) OR ("Ibuprofen"[MeSH Terms] OR "Ibuprofen"[All Fields] OR "ibuprofen s"[All Fields] OR "ibuprofens"[All Fields]) OR ("Ibuprofen"[MeSH Terms] OR "Ibuprofen"[All Fields] OR "motrin"[All Fields] OR "ibuprofen s"[All Fields] OR "ibuprofens"[All Fields]) OR ("Ibuprofen"[MeSH Terms] OR "Ibuprofen"[All Fields] OR "nuprin"[All Fields]) OR ("Ibuprofen"[MeSH Terms] OR "Ibuprofen"[All Fields] OR "rufen"[All Fields]) OR ("Ibuprofen"[MeSH Terms] OR "Ibuprofen"[All Fields]) OR ("Ibuprofen"[MeSH Terms] OR "Ibuprofen"[All Fields] OR "brufen"[All Fields]) OR ("Ibuprofen"[MeSH Terms] OR "Ibuprofen"[All Fields] OR ("trauma"[All Fields] AND "dolgit"[All Fields] AND "gel"[All Fields]) OR "trauma dolgit gel"[All Fields]) OR ("Ibuprofen"[MeSH Terms] OR "Ibuprofen"[All Fields] OR ("trauma"[All Fields] AND "dolgit"[All Fields] AND "gel"[All Fields]) OR "trauma dolgit gel"[All Fields]))) OR ("Prostaglandin Antagonists"[MeSH Terms] OR ("Prostaglandin Antagonists"[Pharmacological Action] OR "Prostaglandin Antagonists"[MeSH Terms] OR ("prostaglandin"[All Fields] AND "antagonists"[All Fields]) OR "Prostaglandin Antagonists"[All Fields] OR ("prostaglandin"[All Fields] AND "antagonist"[All Fields]) OR "prostaglandin antagonist"[All Fields] OR ("Prostaglandin Antagonists"[Pharmacological Action] OR "Prostaglandin Antagonists"[MeSH Terms] OR ("prostaglandin"[All Fields] AND "antagonists"[All Fields]) OR "Prostaglandin Antagonists"[All Fields] OR ("antagonist"[All Fields] AND "prostaglandin"[All Fields]) OR "antagonist prostaglandin"[All Fields]) OR ("Prostaglandin Antagonists"[Pharmacological Action] OR "Prostaglandin Antagonists"[MeSH Terms] OR ("prostaglandin"[All Fields] AND "antagonists"[All Fields]) OR "Prostaglandin Antagonists"[All Fields] OR ("prostaglandin"[All Fields] AND "inhibitor"[All Fields]) OR "prostaglandin inhibitor"[All Fields]) OR ("Prostaglandin Antagonists"[Pharmacological Action] OR "Prostaglandin Antagonists"[MeSH Terms] OR ("prostaglandin"[All Fields] AND "antagonists"[All Fields]) OR "Prostaglandin Antagonists"[All Fields] OR

("inhibitor"[All Fields] AND "prostaglandin"[All Fields]) OR "inhibitor prostaglandin"[All Fields]))) OR ("Acetaminophen"[MeSH Terms] OR ("Acetaminophen"[MeSH Terms] OR "Acetaminophen"[All Fields] OR "paracetamol"[All Fields] OR "acetaminophen s"[All Fields] OR "acetaminophene"[All Fields] OR "acetaminophens"[All Fields] OR "paracetamol s"[All Fields] OR "paracetamols"[All Fields] OR ("Acetaminophen"[MeSH Terms] OR "Acetaminophen"[All Fields] OR "acetominophen"[All Fields]) OR ("Acetaminophen"[MeSH Terms] OR "Acetaminophen"[All Fields] OR "hydroxyacetanilide"[All Fields]) OR ("Acetaminophen"[MeSH Terms] OR "Acetaminophen"[All Fields] OR "paracetamol"[All Fields] OR "acetaminophen s"[All Fields] OR "acetaminophene"[All Fields] OR "acetaminophens"[All Fields] OR "paracetamol s"[All Fields] OR "paracetamols"[All Fields]) OR (("n"[All Fields] AND "4-Hydroxyphenyl"[All Fields]) AND ("acetanilide"[Supplementary Concept] OR "acetanilide"[All Fields] OR "acetanilides"[MeSH Terms] OR "acetanilides"[All Fields])) OR ("Acetaminophen"[MeSH Terms] OR "Acetaminophen"[All Fields] OR "acetamidophenol"[All Fields]) OR ("Acetaminophen"[MeSH Terms] OR "Acetaminophen"[All Fields] OR "n acetyl p aminophenol"[All Fields]) OR ("Acetaminophen"[MeSH Terms] OR "Acetaminophen"[All Fields] OR "acephen"[All Fields] OR "paracetamol"[All Fields] OR "acetaminophen s"[All Fields] OR "acetaminophene"[All Fields] OR "acetaminophens"[All Fields] OR "paracetamol s"[All Fields] OR "paracetamols"[All Fields]) OR ("Acetaminophen"[MeSH Terms] OR "Acetaminophen"[All Fields] OR "acetaco"[All Fields]) OR ("Acetaminophen"[MeSH Terms] OR "Acetaminophen"[All Fields] OR "paracetamol"[All Fields] OR "tylenol"[All Fields] OR "acetaminophen s"[All Fields] OR "acetaminophene"[All Fields] OR "acetaminophens"[All Fields] OR "paracetamol s"[All Fields] OR "paracetamols"[All Fields]) OR ("Acetaminophen"[MeSH Terms] OR "Acetaminophen"[All Fields]) OR ("Acetaminophen"[MeSH Terms] OR "Acetaminophen"[All Fields] OR "datril"[All Fields]) OR ("Acetaminophen"[MeSH Terms] OR "Acetaminophen"[All Fields] OR "panadol"[All Fields] OR "paracetamol"[All Fields] OR "acetaminophen s"[All Fields] OR "acetaminophene"[All Fields] OR "acetaminophens"[All Fields] OR "paracetamol s"[All Fields] OR "paracetamols"[All Fields]) OR ("Acetaminophen"[MeSH Terms] OR "Acetaminophen"[All Fields] OR "acamol"[All Fields]) OR ("Acetaminophen"[MeSH Terms] OR "Acetaminophen"[All Fields] OR "algotropyl"[All Fields]))) OR ("Indomethacin"[MeSH Terms] OR "Indomethacin"[All Fields] OR "indometacin"[All Fields] OR "indomethacine"[All Fields] OR ("Indomethacin"[MeSH Terms] OR "Indomethacin"[All Fields] OR "osmosin"[All Fields]) OR ("Indomethacin"[MeSH

Terms] OR "Indomethacin"[All Fields] OR "indocid"[All Fields]) OR ("Indomethacin"[MeSH Terms] OR "Indomethacin"[All Fields] OR "metindol"[All Fields]) OR ("Indomethacin"[MeSHTerms] OR "Indomethacin"[All Fields] OR "indomet 140"[All Fields]) OR ("Indomethacin"[MeSH Terms] OR "Indomethacin"[All Fields] OR "amuno"[All Fields]) OR ("Indomethacin"[MeSH Terms] OR "Indomethacin"[All Fields] OR "indocin"[All Fields] OR "indometacin"[All Fields] OR "indomethacine"[All Fields]) OR "Indomethacin"[MeSH Terms]))

Figure S1-S4

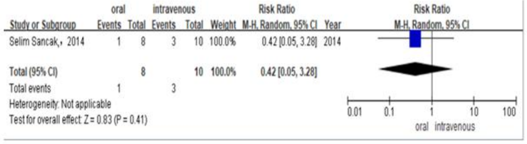

Figure S1 Risk difference of observational studies of Mortality during the paracetamol therapy

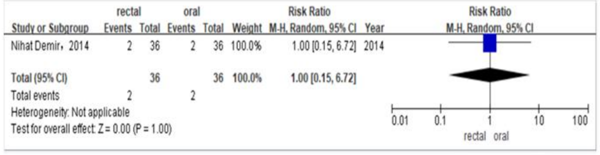

Figure S2 Risk difference of observational studies of Mortality during the ibuprofen therapy

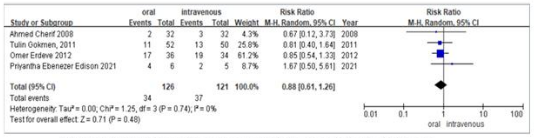

Figure S3 Risk difference of Incidence of BPD/CLD during the ibuprofen therapy

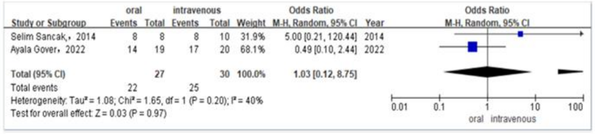

Figure S4 Risk difference of Incidence of BPD/CLD during the paracetamol therapy

Figure S5-S8

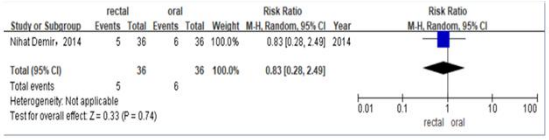

Figure S5 Risk difference of Incidence of BPD/CLD during the ibuprofen therapy

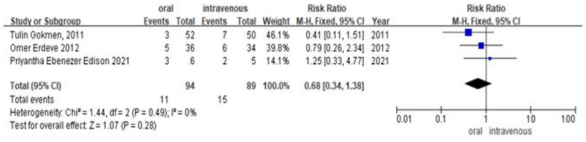

Figure S6 Risk difference of Incidence of ROP during the ibuprofen therapy

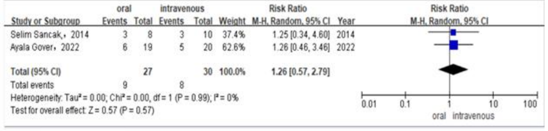

Figure S7 Risk difference of Incidence of ROP during the paracetamol therapy

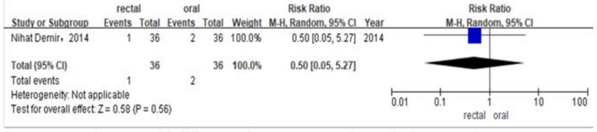

Figure S8 Risk difference of Incidence of ROP during the ibuprofen therapy

Figure S9-S12

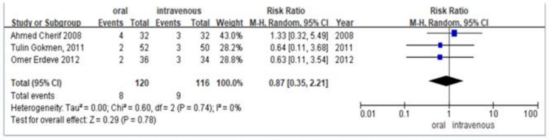

Figure S9 Risk difference of RCTS of Incidence of NEC during the ibuprofen therapy

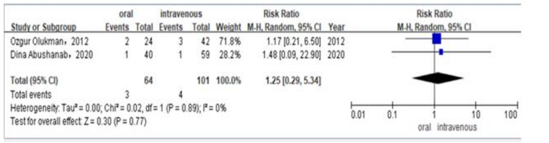

Figure S10 Risk difference of observational studies of Incidence of NEC during the ibuprofen therapy

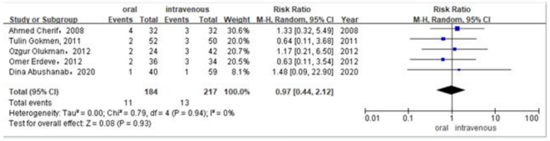

Figure S11 Risk difference of RCTS and observational studies of Incidence of NEC during the ibuprofen therapy

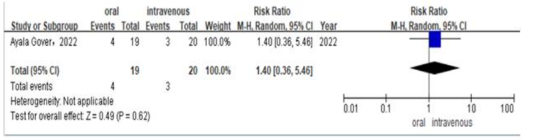

Figure S12 Risk difference of Incidence of NEC during the paracetamol therapy

Figure S13-16

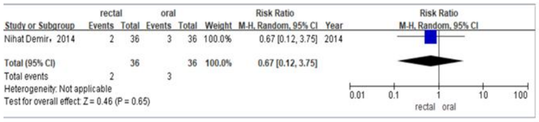

Figure S13 Risk difference of Incidence of NEC during the ibuprofen therapy

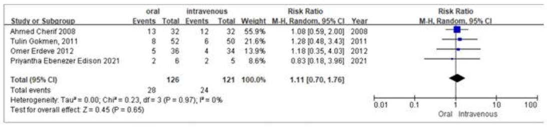

Figure S14 Risk difference of Incidence of IVH during the ibuprofen therapy

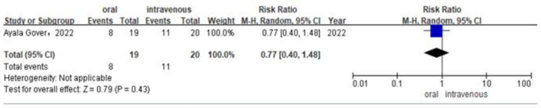

Figure S15 Risk difference of Incidence of IVH during the paracetamol therapy

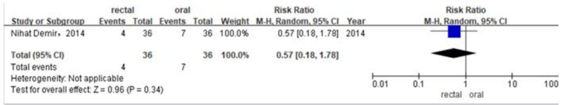

Figure S16 Risk difference of Incidence of IVH during the ibuprofen therapy

Figure S17-S20

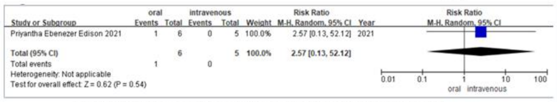

Figure S17 Risk difference of RCTs of Incidence of SIP during the ibuprofen therapy

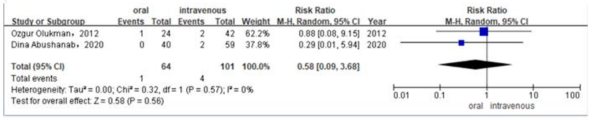

Figure S18 Risk difference of observational studies of Incidence of SIP during the ibuprofen therapy

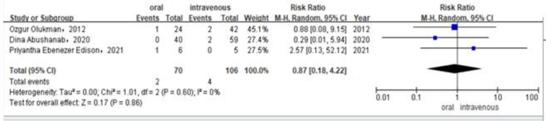

Figure S19 Risk difference of RCTs and observational studies of Incidence of SIP during the ibuprofen therapy

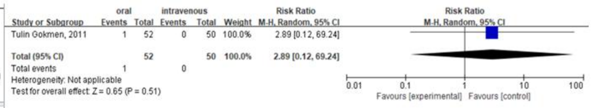

Figure S20 Risk difference of Incidence of GIH during the ibuprofen therapy

Figure S21-24

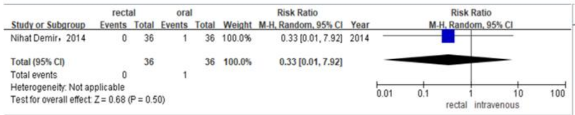

Figure S21 Risk difference of Incidence of GIH during the ibuprofen therapy

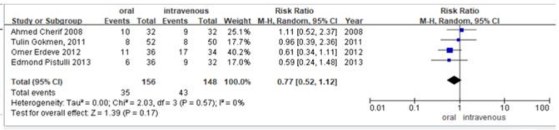

Figure S22 Risk difference of Incidence of Sepsis during the ibuprofen therapy

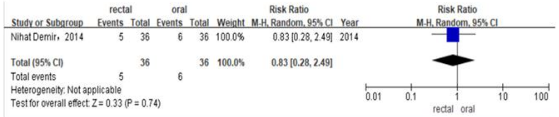

Figure S23 Risk difference of Incidence of Sepsis during the ibuprofen therapy

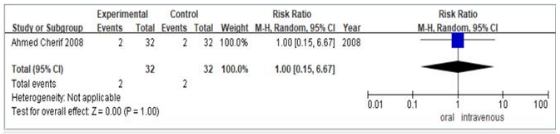

Figure S24 Risk difference of Incidence of PVL during the ibuprofen therapy

Figure S25-28

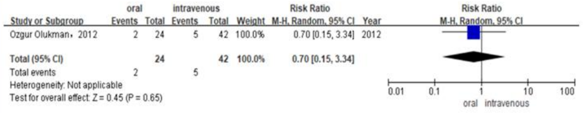

Figure S25 Risk difference of Incidence of oliguria during the ibuprofen therapy

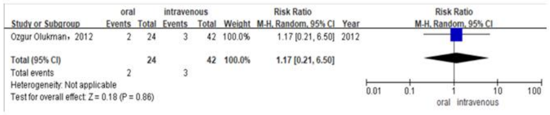

Figure S26 Risk difference of Incidence of pulmonary hemorrhage during the ibuprofen therapy

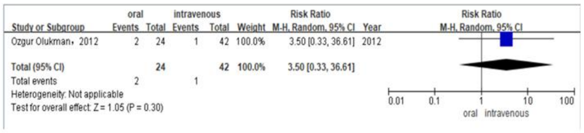

Figure S27 Risk difference of Incidence of pulmonary hypertension during the ibuprofen therapy

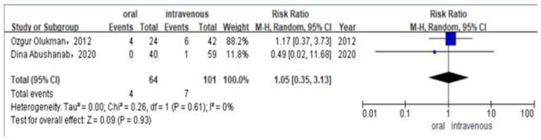

Figure S28 Risk difference of Incidence of thrombocytopenia during the ibuprofen therapy

Figure S29-32

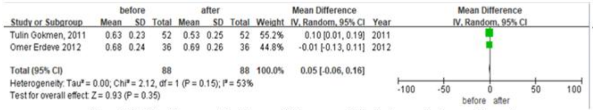

Figure S29 Risk difference of Incidence of Plasma creatinine before and after oral ibuprofen

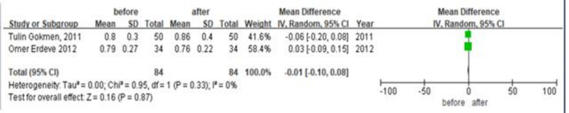

Figure S30 Risk difference of Incidence of Plasma creatinine before and after intravenous ibuprofen

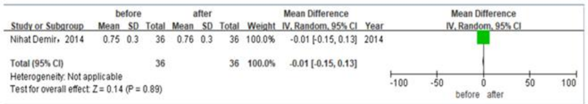

Figure S31 Risk difference of Incidence of Plasma creatinine before and after oral ibuprofen

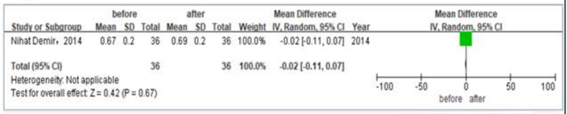

Figure S32 Risk difference of Incidence of Plasma creatinine before and after rectal ibuprofen

Figure S33-S36

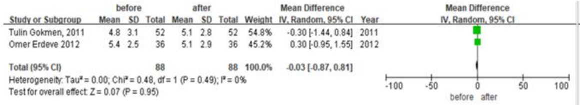

Figure S33 Risk difference of Incidence of plasma bilirubin before and after oral ibuprofen

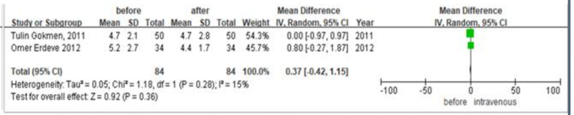

Figure S34 Risk difference of Incidence of plasma bilirubin before and after intravenous ibuprofen

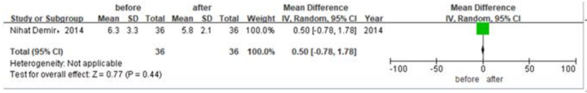

Figure S35 Risk difference of Incidence of plasma bilirubin before and after oral ibuprofen

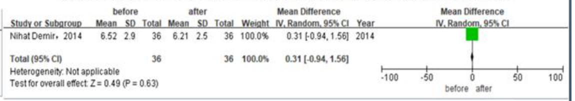

Figure S36 Risk difference of Incidence of plasma bilirubin before and after rectal ibuprofen

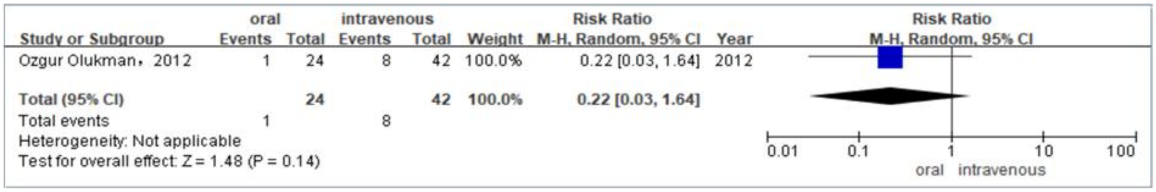

Figure S37 Risk difference of Incidence of hyperbilirubinemia during ibuprofen therapy

FigureA1-FigureA4

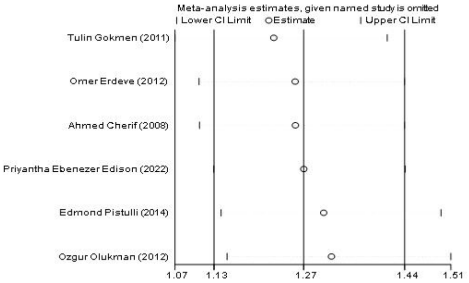

Figure A1 **Meta-analysis estimates** of PDA closure rate after the first course of Ibuprofen

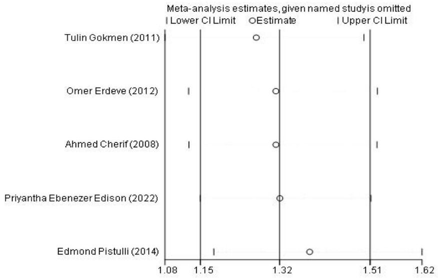

Figure A2 **Meta analysis estimates** of RCTS of PDA closure rate after the first course of Ibuprofen

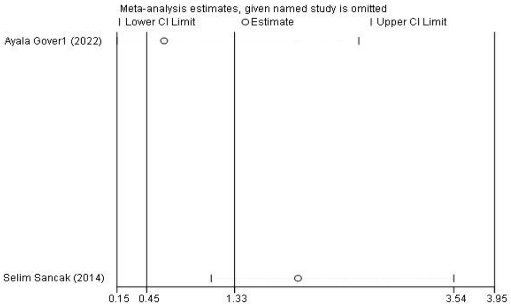

Figure A3 **Meta-analysis estimates** of observational studies of PDA closure rate after the first course of paracetamol

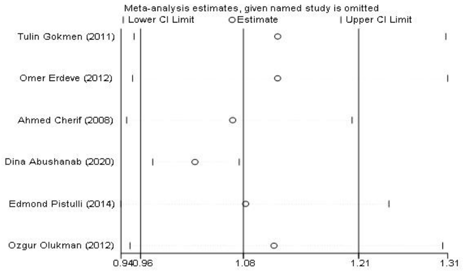

Figure A4 **Meta-analysis estimates** of **combined of RCTs and observational studies** of PDA closure rate after the total course of ibuprofen

FigureA5-FigureA8

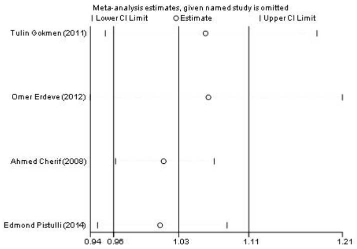

Figure A5 **Meta-analysis estimates** of RCTs after the total course of ibuprofen

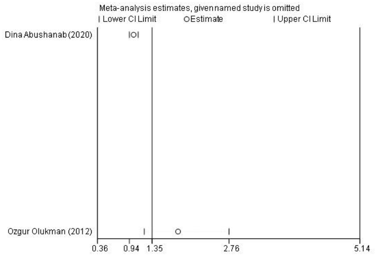

Figure A6 **Meta-analysis estimates** of observational studies after the total course of ibuprofen

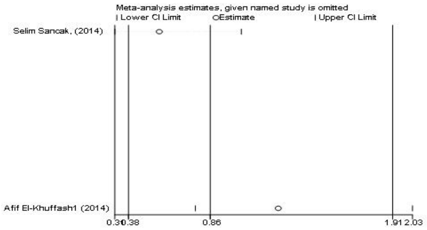

Figure A7 **Meta-analysis estimates** of observational studies after the total course of paracetamol

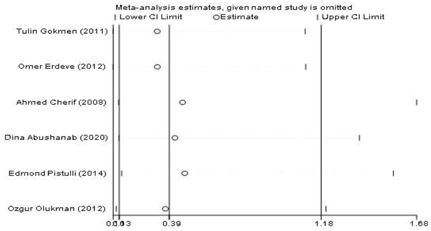

Figure A8 **Meta-analysis estimates** of RCTs and observational studies of need for surgical ligations of PDA after ibuprofen treatment

FigureA9-FigureA12

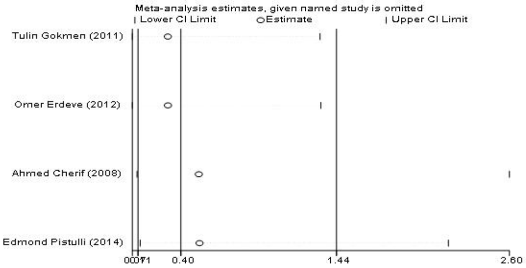

Figure A9 Meta analysis estimates of RCTs of need for surgical ligations of PDA after ibuprofen treatment

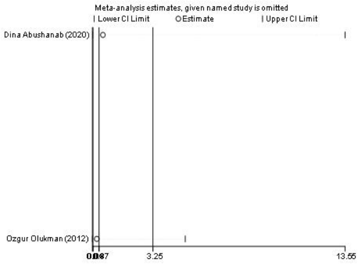

Figure A10 Meta analysis estimates of observational studies of need for surgical ligations of PDA after ibuprofen treatment

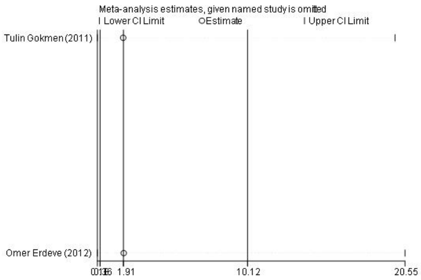

Figure A11 Meta-analysis estimates of RCTs of motality during ibuprofen treatment

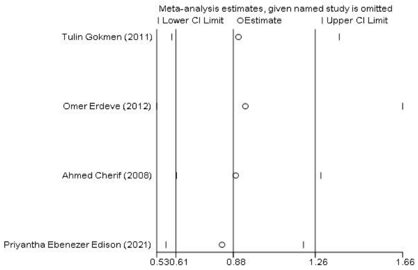

Figure A12 Meta-analysis estimates of incidence of BPD/CLD during ibuprofen treatment

FigureA13-FigureA16

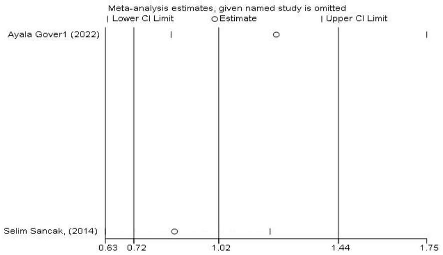

Figure A13 Meta-analysis estimates of incidence of BPD/CLD during paracetamol treatment

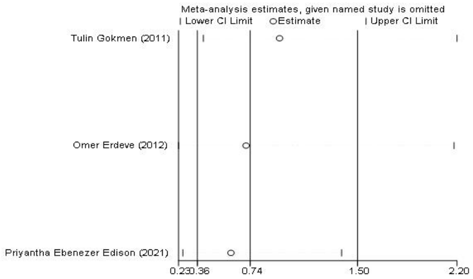

Figure A14 Meta-analysis estimates of incidence of ROP during ibuprofen treatment

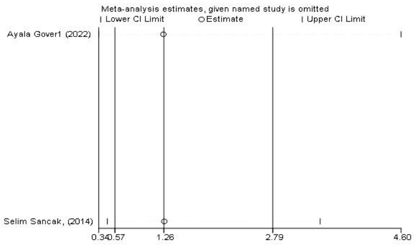

Figure A15 Meta-analysis estimates of incidence of ROP during paracetamol treatment

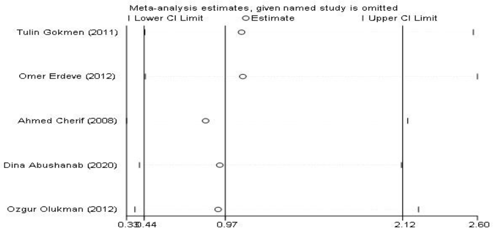

Figure A16 Meta-analysis estimates of RCTs and observational studies of incidence of NEC during ibuprofen treatment

FigureA17-FigureA20

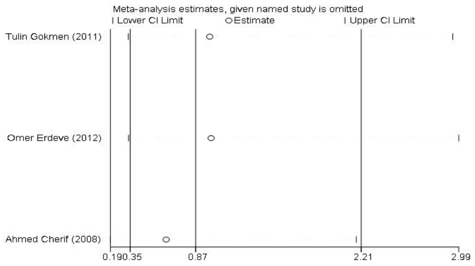

Figure A17 meta-analysis estimates of RCTs of incidence of NEC during ibuprofen treatment

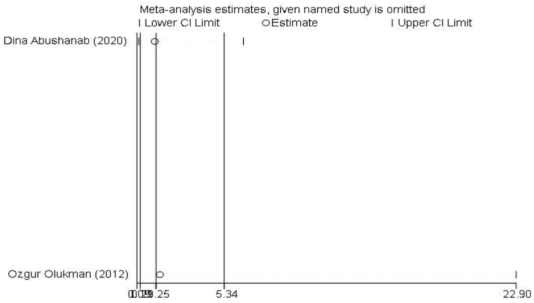

Figure A18 Meta-analysis estimates of observational studies of incidence of NEC during ibuprofen treatment

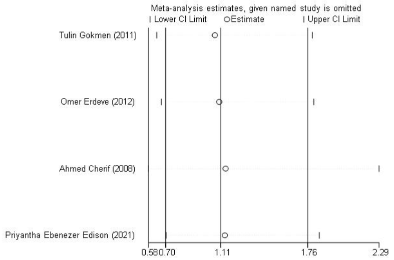

Figure A19 Meta-analysis estimates of incidence of IVH during ibuprofen treatment

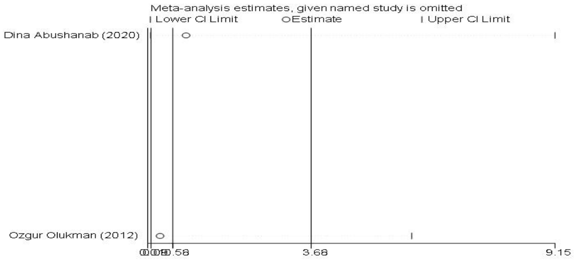

Figure A20 Meta-analysis estimates of observational study of incidence of SIP during ibuprofen treatment

FigureA21-FigureA24

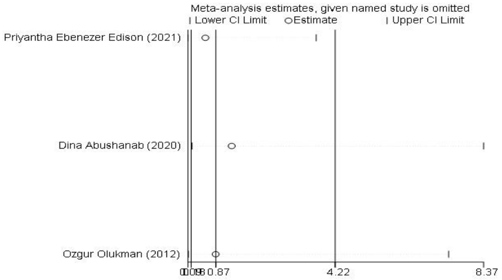

Figure A21 Meta-analysis estimates of RCTs and observational study of incidence of SIP during ibuprofen treatment

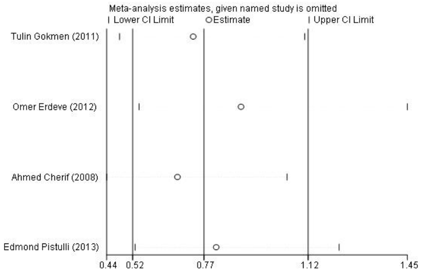

Figure A22 Meta-analysis estimates of incidence of Sepsis during ibuprofen treatment

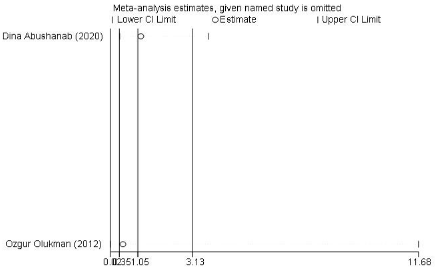

Figure A23 Meta-analysis estimates of incidence of thrombocytopenia during ibuprofen treatment

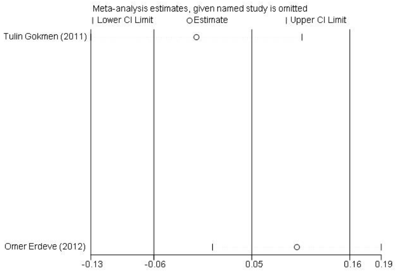

Figure A24 Meta-analysis estimates of plasma creatinine before and after oral ibuprofen

FigureA25-FigureA27

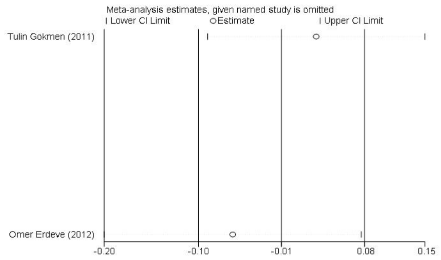

Figure A25 Meta-analysis estimates of plasma creatinine before and after intravenous ibuprofen

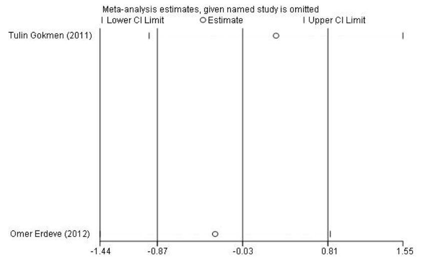

Figure A26 Meta-analysis estimates of plasma bilirubin before and after oral ibuprofen

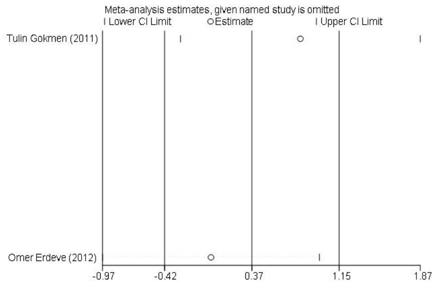

Figure A27 Meta-analysis estimates of plasma bilirubin before and after intravenous ibuprofen

FigureB1-FigureB4

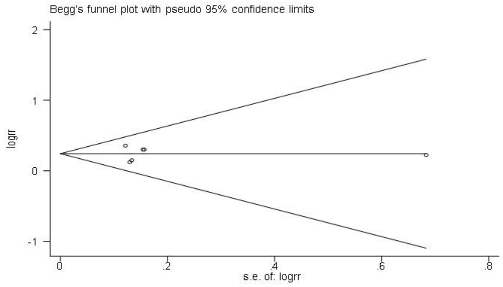

Figure B1 Begg's funnel plot for PDA closure rate after the first course of Ibuprofen

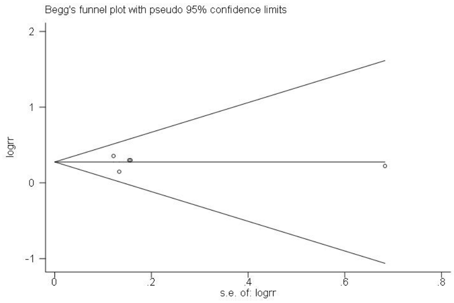

Figure B2 Begg's funnel plot for RCTS of PDA closure rate after the first course of Ibuprofen

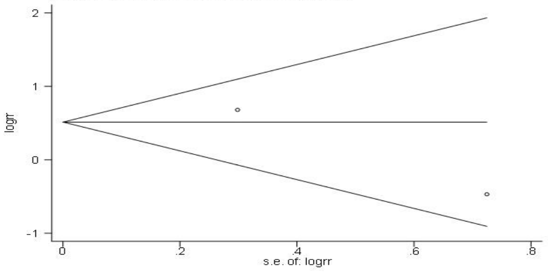

Figure B3 Begg's funnel plot for observational studies of PDA closure rate after the first course of paracetamol

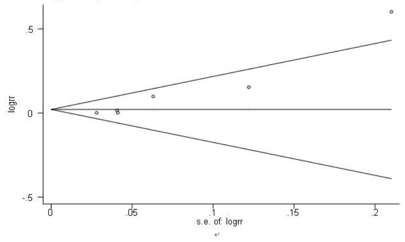

Figure B4 Begg's funnel plot for combined of RCTs and observational studies of PDA closure rate after the total course of ibuprofen

FigureB5-FigureB8

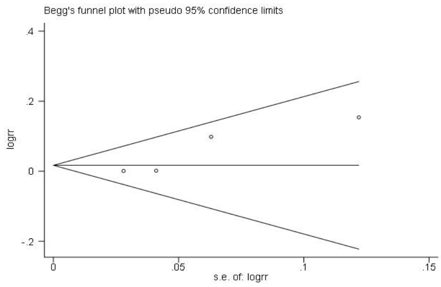

Figure B5 Begg’s funnel plot for RCTs after the total course of ibuprofen

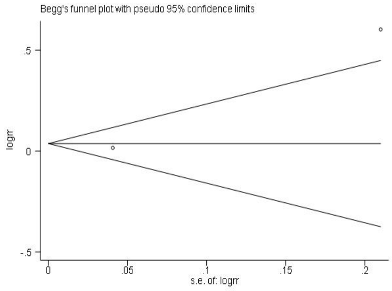

Figure B6 Begg’s funnel plot for observational studies after the total course of ibuprofen

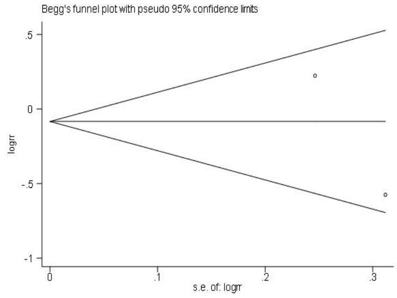

Figure B7 Begg’s funnel plot for observational studies after the total course of paracetamol

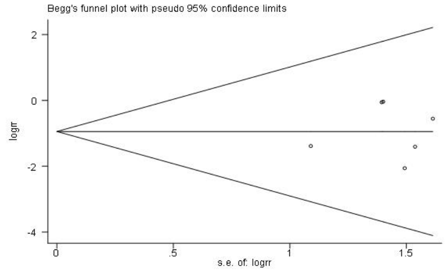

Figure B8 Begg’s funnel plot for RCTs and observational studies of need for surgical ligations of PDA after ibuprofen treatment

FigureB9-FigureB12

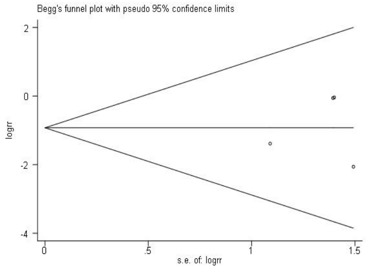

Figure B9 Begg’s funnel plot for RCTs of need for surgical ligations of PDA after ibuprofen treatment

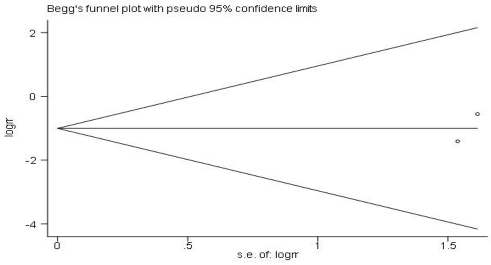

Figure B10 Begg’s funnel plot for observational studies of need for surgical ligations of PDA after ibuprofen treatment

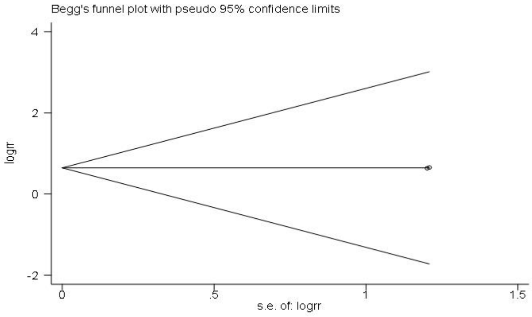

Figure B11 Begg’s funnel plot for RCTs of motality during ibuprofen treatment

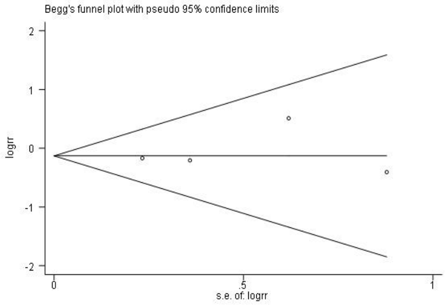

Figure B12 Begg’s funnel plot for incidence of BPD/CLD during ibuprofen treatment

FigureB13-FigureB16

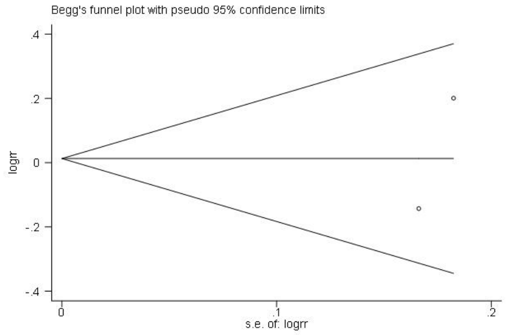

Figure B13 Begg’s funnel plot for incidence of BPD/CLD during paracetamol treatment

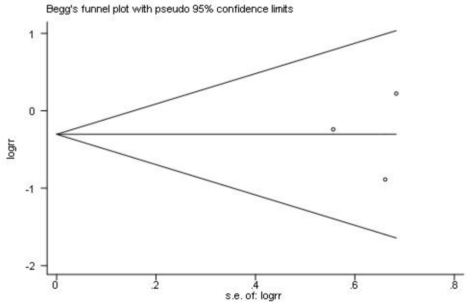

Figure B14 Begg’s funnel plot for ROP during ibuprofen treatment

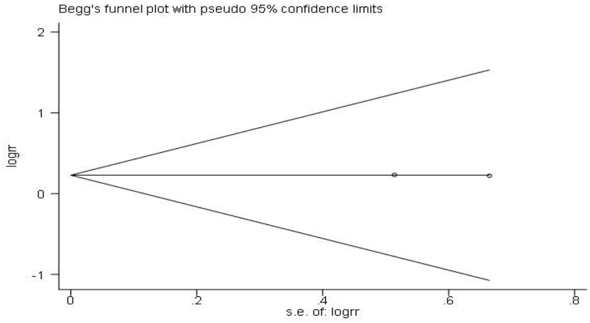

Figure B15 Begg’s funnel plot for ROP during paracetamol treatment

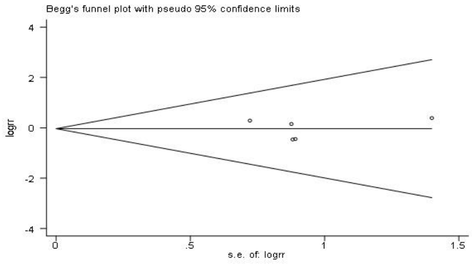

Figure B16 Begg’s funnel plot for RCTs and observational studies of incidence of NEC during ibuprofen treatment

FigureB17-FigureB20

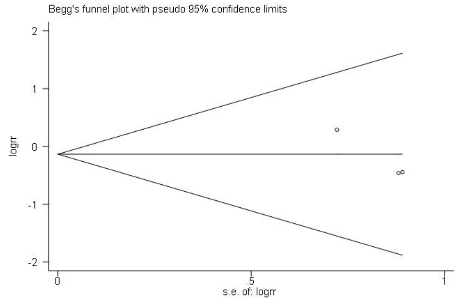

Figure B17 Begg’s funnel plot for RCTs of incidence of NEC during ibuprofen treatment

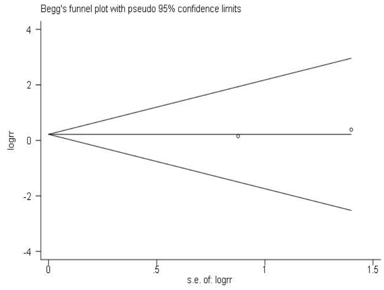

Figure B18 Begg’s funnel plot for observational studies of incidence of NEC during ibuprofen treatment

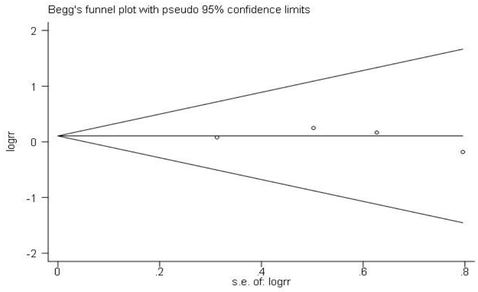

Figure B19 Begg’s funnel plot for incidence of IVH during ibuprofen treatment

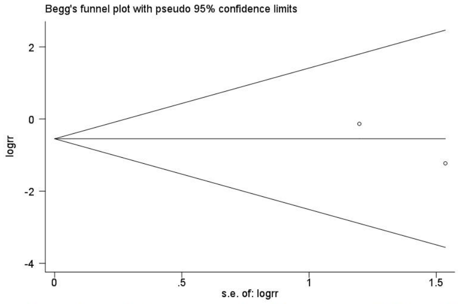

Figure B20 Begg’s funnel plot for observational study of incidence of SIP during ibuprofen treatment

FigureB21-FigureB24

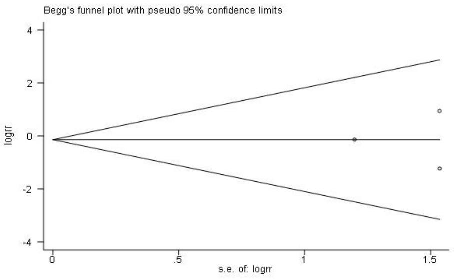

Figure B21 Begg' s funnel plot for RCTs and observational study of incidence of SIP during ibuprofen treatment

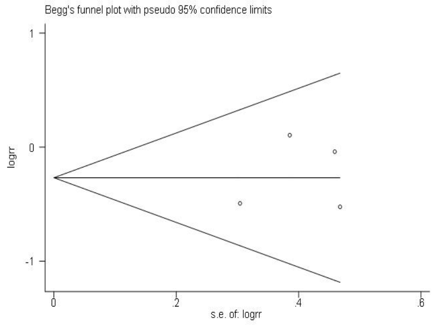

Figure B22 Begg' s funnel plot for incidence of Sepsis during ibuprofen treatment

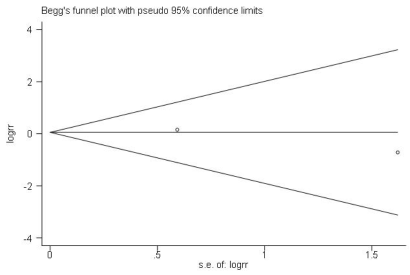

Figure B23 Begg' s funnel plot for incidence of thrombocytopenia during ibuprofen treatment

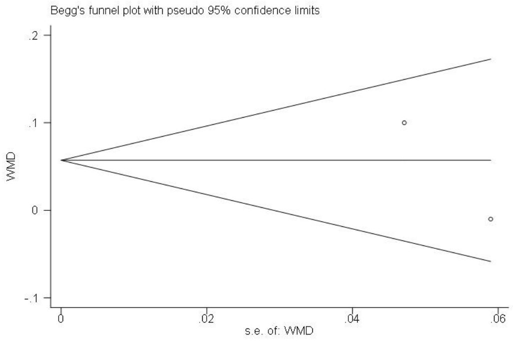

Figure B24 Begg' s funnel plot for plasma creatinine before and after oral ibuprofen

FigureB25-FigureB27

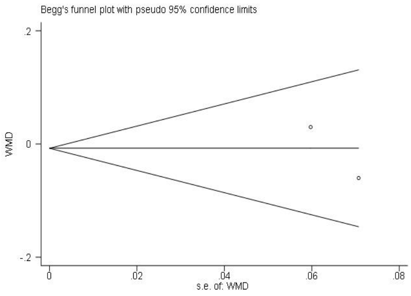

Figure B25 Begg' s funnel plot for plasma creatinine before and after intravenous ibuprofen

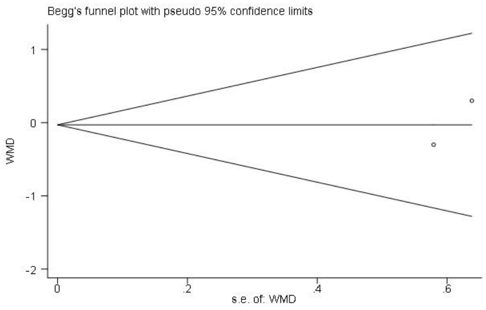

Figure B26 Begg' s funnel plot for plasma bilirubin before and after oral ibuprofen

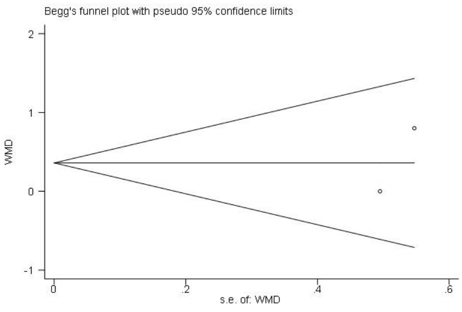

Figure B27 Begg' s funnel plot for plasma bilirubin before and after intravenous ibuprofen
