# Supplementary material for: The impact of the route of administration on the efficacy and safety of the drug therapy for patent ductus arteriosus in premature infants: a systematic review and meta-analysis
Source: PeerJ. 2024 Jan 29;12:e16591. doi: 10.7717/peerj.16591 (PMC10832619; doi:10.7717/peerj.16591)
Supplement: Supplemental Information 3 [file peerj-12-16591-s003.docx]

**Comment**

**Systematic Review Registration**

Please explain why formal screening of search results against eligibility criteria began before submission to PROSPERO.

**Reply:** We would like to express our heartfelt gratitude for your instructive suggestions. We have reviewed our project registration program again. Prospero is prospective registration platform, so we should start the registration before starting our study. If we start registering when we extract data, the registration will automatically be refused by Prospero, because it clearly does not meet the requirement of prospective registration of Prospero. Prospero registration include 6 stages: 1 Preliminary searches; 2 Piloting of the study selection process; 3 Formal screening of search results against eligibility criteria; 4 Data extraction; 5 Risk of bias; 6 (quality) assessment; 7 Data analysis. We have completed the first and second steps before registration, and start working on the third step. Before registration, we conducted a preliminary search to determine the research direction and a part of the preliminary screening work (not yet completed). On the one hand, we want to comprehensively evaluate this topic to ensure that the research can be carried out effectively. In addition, we conduct preliminary screening before registration to grasp more details of our follow-up research, so that we can reduce deviation between our planned study and the actual content of subsequent manuscript, making it easier for readers to understand our article.
