# Supplementary material for: The impact of the route of administration on the efficacy and safety of the drug therapy for patent ductus arteriosus in premature infants: a systematic review and meta-analysis
Source: PeerJ. 2024 Jan 29;12:e16591. doi: 10.7717/peerj.16591 (PMC10832619; doi:10.7717/peerj.16591)
Supplement: Supplemental Information 4 [file peerj-12-16591-s004.docx]

## Systematic Review and/or Meta-Analysis Rationale

For systematic reviews / meta-analyses, authors need to provide the following information:

**1.Comment:** The rationale for conducting the systematic review / meta-analysis.

**Responds:** This systematic review and meta-analysis aims to explore the potential impact of the route of administration on efficacy and adverse events when medications are administered to premature infants with PDA. By investigating the relationship between routes of administration and treatment outcomes, we hope to gain insights into how to optimize the drug therapy of PDA in preterm infants, while minimizing adverse effects. Compared with previous meta-analyses and analysis, RCTs and observational studies with more cases were included in our study. It showed that oral ibuprofen or paracetamol owned their priority than the intravenous ones to a certain extent regarding closing PDA without increasing the risk of adverse events. Our meta-analysis demonstrated that oral ibuprofen and acetaminophen were associated with minimal adverse effects when used for PDA closure and that premature infants can tolerate oral preparation well.

**2.Comment:** The contribution that it makes to knowledge in light of previously published related reports, including other meta-analyses and systematic reviews.

**Responds:** Failure of ductus closure (PDA) is common in premature infants If the ductus remains open, persistent and substantial left-to-right shunting via the ductus could lead to pulmonary circulation blood stasis, systemic circulation ischemia, and a series of complications such as renal, gastrointestinal, and cerebral effects. The first-line medications for treating hsPDA are non-selective cyclooxygenase (COX) inhibitors. However, some studies have reported adverse events associated with the use of COX and POX inhibitors. This systematic review and meta-analysis was conducted to investigate the potential impact of the route of drug administration on the efficacy and adverse events when medications are applied to premature infants with PDA.
